# Importance of Coping Strategies on Quality of Life in People with Multiple Sclerosis: A Systematic Review

**DOI:** 10.3390/jcm13185505

**Published:** 2024-09-18

**Authors:** Laura Culicetto, Viviana Lo Buono, Sofia Donato, Antonino La Tona, Anita Maria Sophia Cusumano, Graziana Marika Corello, Edoardo Sessa, Carmela Rifici, Giangaetano D’Aleo, Angelo Quartarone, Silvia Marino

**Affiliations:** 1 IRCCS Centro Neurolesi “Bonino-Pulejo”, S.S. 113 Via Palermo C. da Casazza, 98124 Messina, Italy; laura.culicetto@irccsme.it (L.C.); edoardo.sessa@irccsme.it (E.S.); carmela.rifici@irccsme.it (C.R.); giangaetano.daleo@irccsme.it (G.D.); angelo.quartarone@irccsme.it (A.Q.); silvia.marino@irccsme.it (S.M.); 2 Department of Clinical and Experimental Medicine, University of Messina, 98122 Messina, Italy; sofia.donato@studenti.unime.it; 3 Department of Human and Social Sciences, University of Bergamo, 24129 Bergamo, Italy; antonino.latona@unibg.it; 4 Dipartimento di Scienze Psicologiche, Pedagogiche, Dell’esercizio Fisico e Della Formazione, Università degli Studi di Palermo, 90133 Palermo, Italy; anitamariasophia.cusumano@community.unipa.it; 5 Department of Science of Education, Section of Psychology, University of Catania, 95124 Catania, Italy; grazianacorello@live.it

**Keywords:** coping strategies, multiple sclerosis, quality of life, rehabilitation, sociodemographic variables, emotional factors

## Abstract

Multiple sclerosis (MS) is a neurodegenerative disorder of the central nervous system characterized by a variety of symptoms such as fatigue, spasticity, tremors, and cognitive disorders. Individuals with MS may employ different coping strategies to manage these symptoms, which in turn can significantly impact their quality of life (QoL). This review aims to analyze these coping strategies and their impact on QoL. Furthermore, it seeks to identify the key factors that influence the choice and effectiveness of these coping strategies, providing insights into which strategies are most beneficial for enhancing QoL in people with MS. **Methods:** Systematic searches were performed in Scopus, PubMed, Web of Science, and Scopus databases. This systematic review has been registered in OSF with the number DOI 10.17605/OSF.IO/QY37X. **Results**: A total of 1192 studies were identified. After reading the full text of the selected studies and applying predefined inclusion criteria, 19 studies were included based on their pertinence and relevance to the topic. The results revealed that emotional variables, demographic factors, personality traits, and family support significantly influence the choice of coping strategies used to manage the symptoms of MS. Problem-solving and task-oriented coping were prevalent among MS patients and associated with better QoL outcomes. Emotional-focused and avoidance strategies were generally linked to poorer QoL, though avoidance provided temporary relief in certain contexts. Social support, emotional health, and cognitive reframing were crucial in enhancing QoL. **Conclusions**: The findings underscore the importance of tailored psychoeducational and therapeutic interventions focusing on emotional health, social support, and adaptive coping strategies. These interventions can significantly improve the long-term outcomes for individuals with MS. Future research should explore the dynamic interactions between coping strategies and QoL over time, providing a comprehensive understanding of how to best support MS patients in managing their disease.

## 1. Introduction

Multiple sclerosis (MS) is a chronic and neurodegenerative disorder of the central nervous system characterized by several symptoms that can occur individually or together, either as sudden episodes or as part of a gradual progression [1]. Common initial symptoms include tingling, numbness, muscle weakness, visual disturbances, double vision, and coordination problems. Other symptoms may include fatigue, spasticity, balance issues, sensory loss, pain, urinary and sexual dysfunction, depression, cognitive difficulties, and heat intolerance [2,3] The 2017 revisions to the McDonald criteria, presented by the International Panel on Diagnosis of multiple sclerosis, introduced a key change from the 2010 criteria. Now, symptomatic lesions are included in the total count of demyelinating lesions. This means that a diagnosis of primary progressive multiple sclerosis can be made with just one symptomatic lesion in the infratentorial, periventricular, cortical, or juxtacortical areas, provided there is at least one year of disease progression and evidence of cerebrospinal fluid oligoclonal bands [4]. The prevalence of MS ranges from 15 to 250 per 100,000 individuals, corresponding to approximately 0.015% to 0.25% of the population. It is estimated that two million people worldwide are affected by MS [5]. This estimate can vary. For example, in Italy, the estimated prevalence of MS is approximately 193 cases per 100,000 inhabitants, which equates to 0.193% of the population. In Sardinia, however, the prevalence is higher, at 360 cases per 100,000 inhabitants, representing 0.36% of the population [6]. Women are affected by MS two to three times more often than men, with the condition primarily impacting individuals between the ages of 20 and 40 years [7,8].

Phisycal disability and cognitive impairment, associated with the unpredictability of the progression of symptoms with periods of remission and relapse, could affect the everyday well-being and health-related quality of life (HRQoL) of patients [9,10]. HRQoL in MS is not only recognized as a crucial gauge of disease impact but also as a predictor of disease progression [11,12] and appears to correlate with surrogate markers such as lesion burden and brain volume measures on magnetic resonance imaging (MRI) [13]. The impact of MS depends on several factors, particularly age, educational level, marital status [14], disability level, fatigue [15], social support [16], and cognitive appraisals.

Quality of life (QoL) is the perception that people have about their life in relation to their cultural and moral context [17]. Factors such as the stigmatization of MS and the variety of symptoms like pain and fatigue produce changes in social life with positive effects such as family support and negative effects like miscomprehension [18].

People often develop routine methods to handle stressors or utilize coping strategies that are tailored to the specific type of stressful situation [19]. Coping style is a modifiable factor influencing psychological well-being in the general population throughout the lifespan [20,21]. Coping strategies involve cognitive and behavioral efforts aimed at minimizing and managing external and internal demands that individuals perceive as taxing or overwhelming [22]. Coping can be considered a response to a present or past frustrating situation or in anticipation of a stressful confrontation [23] and can be classified as active/adaptive or avoidant/maladaptive [24]. Active coping entails employing adaptive strategies that facilitate overcoming stress and achieving a healthy or desired state (e.g., action-oriented approaches). Conversely, avoidant coping consists of less adaptive strategies (e.g., denial, behavioral, and mental disengagement).

There are three types of coping strategies, such as problem-focused, which includes solving, reconceptualizing, or minimizing the effects of stressors; emotion-focused, including affect regulation; and avoidant, which refers to wishful thinking, escapism, and efforts to distract oneself [25].

There is evidence regarding the influence of cognitive abilities in MS on coping strategies [26]. In general, a higher cognitive reserve seems to exert a protective effect, shielding against the onset of depression, cognitive impairment, and reduced QoL in MS [27,28].

Generally, adaptive coping strategies are linked to improved physical and mental health outcomes [29,30], while avoidant coping strategies tend to be associated with poorer physical and mental health outcomes [24,31] and reduced QoL [32]. It was shown that the flexible fusion of problem- and emotion-focused strategies could be considered “adaptive coping” [33].

In light of the existing literature, we hypothesized that adaptive coping strategies would be associated with better QoL in individuals with MS, whereas maladaptive coping strategies would correlate with poorer QoL outcomes. Additionally, we anticipated that certain demographic and psychosocial factors, such as age, gender, social support, and cognitive reserve, would significantly influence the choice of coping strategies.

With these considerations in mind, the aim of this study was to examine the impact of coping strategies employed by people with MS on their QoL and to identify the factors, such as psychological and social dimensions, that influence these coping strategies, determining which strategies are most effective. Additionally, our goal was to determine which coping strategies are most effective in enhancing the well-being of MS patients across different stages of the disease, thereby providing actionable insights for healthcare professionals in developing targeted interventions that support adaptive coping mechanisms.

## 2. Materials and Methods

**Figure 1:**
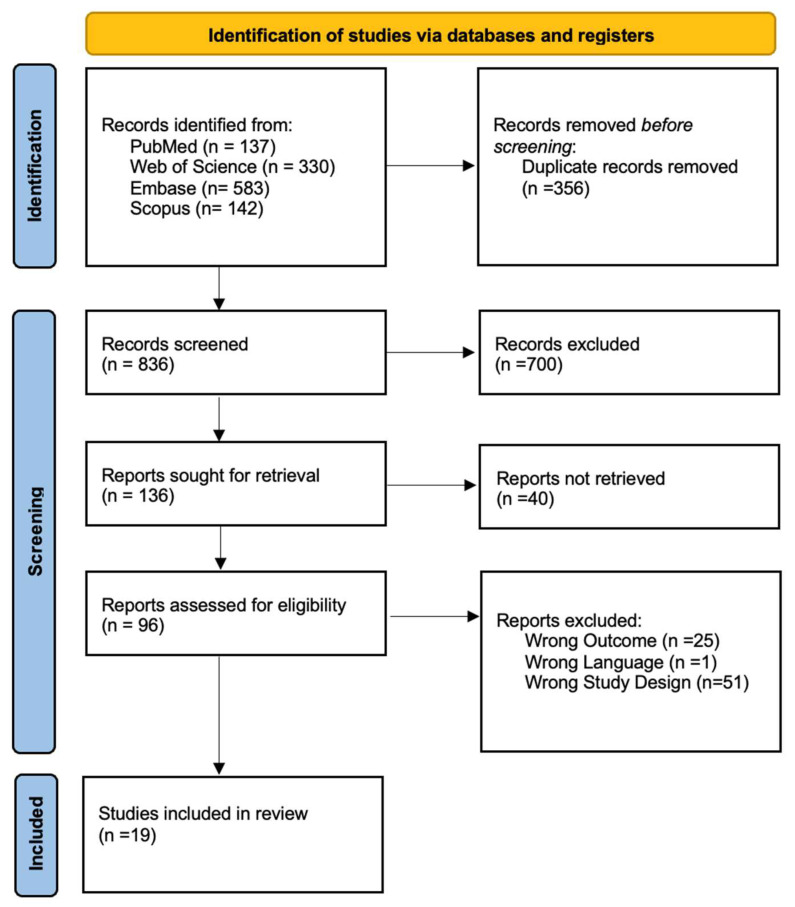
PRISMA Flow Chart.

This systematic review was conducted and reported in accordance with the Preferred Reporting Items for Systematic Review and Meta-Analyses (PRISMA) (see Figure 1) [34]. A protocol for this review was registered on OSF (DOI 10.17605/OSF.IO/QY37X) [35].

### 2.1. Search Strategy

The studies were identified by searching in Scopus, PubMed, Web of Science, and Scopus databases in April 2024. All the studies fulfilling our selected criteria were evaluated for possible inclusion. The search combined the following terms: (“coping strategies” OR “coping skills” OR “adaptation strategies”) AND (“multiple sclerosis” OR “MS”) AND (“quality of life” OR “life quality” OR “well-being”).

The search terms were identified for title and abstract. After duplicates had been removed using EndNote, the title and abstract screening, as well as the full-text screening, were conducted on Rayyan. This research was not restricted by the year of publication for the articles considered.

To shape our research question and guide the selection of studies for this systematic review, we employed the PICO (Population, Intervention, Comparison, and Outcome) [36]. Our target population comprises adults (>18 years) affected by MS. The intervention involves the use of various coping strategies. For the comparison, we focused on patients who employ adaptive coping strategies to those who rely on maladaptive strategies. The outcome is the impact of coping strategies on the QoL, as measured by standardized tests. Our research explores the most effective coping strategies for managing MS symptoms at various stages of the disease and their impact on QoL.

In line with this framework, our inclusion criteria were set to identify studies that assessed both coping strategies and QoL in the context of MS and articles in English language. We excluded reviews and meta-analyses as well as duplicated studies, single case studies, and conference proceedings.

### 2.2. Study Selection

To minimize bias and ensure a robust selection process, two authors (L.C. and V.L.B.) independently reviewed and extracted data from the studies. Any discrepancies were resolved through collaborative discussion and consultation with a third author (S.D.). This multi-step approach guaranteed that at least three researchers independently assessed each article. In cases of persistent disagreement, all authors were involved in the final decision.

### 2.3. Data Extraction and Analysis

The studies that met the inclusion criteria were summarized based on the following points: (1) Study characteristics: type of study and the country in which the data had been collected; (2) Patient characteristics: the sample size, age, gender, duration of disease, and education; (3) Instruments utilized for measuring coping strategies and quality of life; and (4) main and relevant findings. Moreover, the agreement between the two reviewers (L.C. and V.L.B.) was assessed using the kappa statistic. The kappa score, with an accepted threshold for substantial agreement set at >0.61, was interpreted to reflect substantial concordance between the reviewers. This criterion ensures a robust evaluation of the inter-rater reliability, emphasizing the achievement of a substantial level of agreement in the data extraction process.

### 2.4. Risk of Bias within Individual Studies

**Figure 2:**
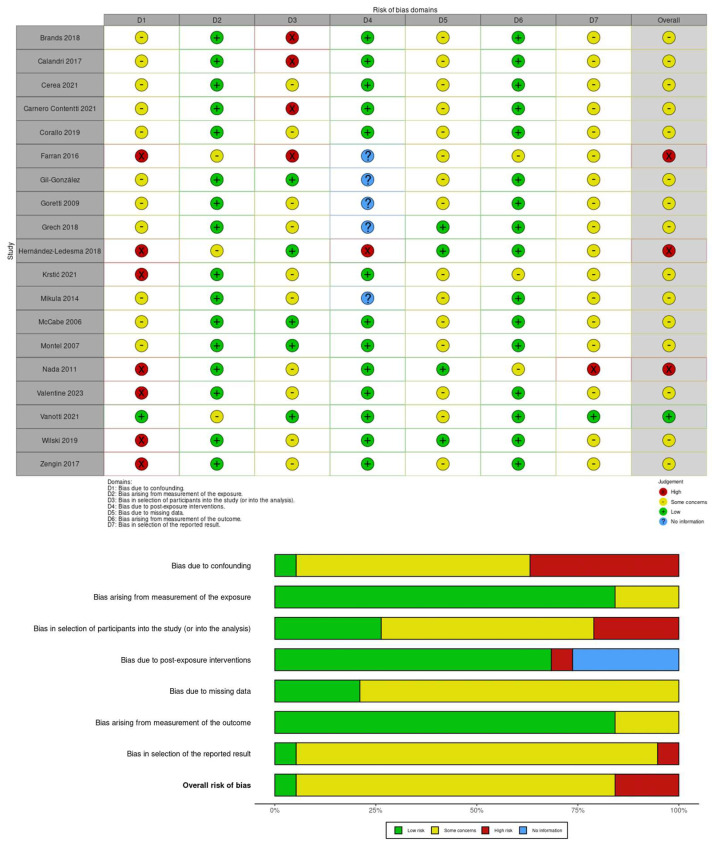
Shows the Risk of Bias (ROBINS-E) of studies regarding the coping strategies used in MS and their impact on QoL [23,32,36,37,38,39,40,41,42,43,44,45,46,47,48,49,50,51,52].

The risk of bias in the selected studies was independently assessed by L.C. and V.L.B., and any disagreements during this process, as well as during previous stages, were resolved through consultation with S.D., who provided the final decision. We used the revised Cochrane tool for non-randomized controlled studies-of exposures (ROBINS-E) tool (Figure 2), which comprises seven domains: (i) bias due to confounding, (ii) bias arising from measurement of the exposure, (iii) bias in selection of participants into the study (or into the analysis), (iv) bias due to post-exposure interventions, (v) bias due to missing data, (vi) bias arising from measurement of the outcome, and (vii) bias in selection of reported result.

## 3. Results

### 3.1. Synthesis of Evidence

A total of 1192 articles were identified through database searches. Following the removal of duplicates, 836 studies were screened by title and abstract. Following full text selection, 19 studies were included for analysis. The selection process is shown in Figure 1.

### 3.2. Key Findings from Included Studies

The studies reviewed offer a comprehensive insight into how coping strategies affect the QoL in individuals with MS (Table 1). Findings emphasize the significance of emotional variables, demographic factors, and personality traits in managing MS effectively [36,37,38].

There is a consensus among the studies that problem-focused and task-oriented coping strategies are generally associated with better QoL outcomes. For example, problem-solving and seeking social support were commonly linked to higher QoL, particularly in the mental health domain [36,38,39,40]. On the other hand, avoidant coping strategies, such as denial and emotional preoccupation, tend to correlate with lower QoL and increased psychological distress, including depression and anxiety [41,42,43].

Some studies also pointed out that demographic and clinical characteristics also influence coping strategies. Women, older patients, and those with progressive MS tend to use denial and religion-based coping [44]. Conversely, individuals with higher education levels were more inclined towards adaptive coping mechanisms like problem-solving and seeking emotional support [44,45]. There is general agreement among the studies that affective factors significantly influence coping choices. Higher depression scores lead to more avoidance strategies, negatively impacting QoL, while anxiety also detracts from mental QoL domains [23]. This is further supported by findings from the Mexican MS population, where negative psychological factors like depression and anxiety were found to adversely affect QoL, underscoring the need for effective coping mechanisms and strong psychosocial support [46]. Additionally, patients with secondary progressive MS (SPMS) who experience higher anxiety and depression tend to use more emotional coping strategies and report lower QoL [42]. Conversely, positive reinterpretation and growth can enhance QoL by reducing stress, depression, and anxiety, whereas maladaptive strategies like emotional preoccupation worsen these outcomes [43,47].The studies show a consistent broad agreement that coping strategies have a significant impact on the relationship between daily pain, fatigue fluctuations, and the resulting functional and emotional outcomes in MS patients [40,41,48]. Avoidant coping strategies lead to greater functional and emotional difficulties, while approach strategies are associated with fewer challenges and better treatment adherence, particularly for injectable treatments [49]. Moreover, personality traits play a crucial role in MS management; neuroticism is linked to negative health outcomes and passive coping strategies, reducing QoL [50]. Additionally, it was noted that [51] MS patients use less effective problem-solving strategies and score higher in traits like psychoticism compared to controls, with no correlation between personality traits and subcortical atrophy [51]. A longitudinal study further supports these findings, reporting that positive coping strategies and social support predict better QoL, with the relationship between coping strategies and QoL domains remaining stable over time, highlighting the enduring influence of coping on QoL [32].

### 3.3. Risk of Bias

The Risk Of Bias In Non-randomized Studies—of Exposures (ROBINS-E) tool was used to assess the risk of bias of the articles included in this review. Figure 2 shows the summary of the risk of bias assessment. Out of the total studies assessed, only one [53] showed low risk of bias due to confounding, seven displayed high risk [37,45,46,47,48,50,51] (30%), and eleven had some concerns [23,32,36,39,40,41,42,43,44,49,52] (60%). Moreover, only three studies (15%) showed some concerns about bias arising from measurement of the exposure [38,46,47]. Further, four studies (20%) exhibited a high risk of bias in the selection of participants into the study [36,41,47,52], and ten (60%) showed some concerns [23,37,39,40,43,45,48,49,50,51]. One study showed a high risk of bias due to post-exposure interventions [46]. All studies, except four (20%) [37,43,46,51], reported some concerns about the bias due to missing data (80%). In contrast, all studies selected reported a low risk of bias arising from the measurement of the outcome except three (15%) [47,50,51]. Additionally, all studies reported some concerns about bias in the selection of the reported result (80%), except one that showed a high risk [51] and another that reported a low risk [38].

## 4. Discussion

The aim of this review is to analyze coping strategies in MS and their impact on QoL. The studies analyzed report that numerous factors intersect with the use of coping strategies, such as demographic variables, emotional factors, social support, and severity of symptoms, with influences on QoL.

**Emotional factors**

MS patients who frequently engage in avoidance coping report lower emotional distress and improved QoL [36]. While this may seem counterintuitive, it’s important to note that in the Coping Inventory for Stressful Situations, avoidance involves actively seeking social support and distractions, unlike its typical association with passive and unproductive behaviors in many other coping assessments. This result aligns with findings from Mikula et al. [40], which indicate that problem-focused coping leads to successful adaptation in MS.

A study [42] reported the strong relation between disease progression and its impact on emotions (depression, mood, anxiety) and QoL. Particularly, patients with SPMS had higher depression and anxiety levels and lower QoL scores. Conversely, patients with PPMS had lower depression and anxiety scores and a better QoL. The precarious condition of SPMS patients can be attributed to the cycle of relapses and recoveries, while primary progressive multiple sclerosis (PPMS) patients have adapted to constant physical impairments from disease onset. Other findings also indicate that the duration of the disease does not exacerbate depression, supporting the theory that MS patients develop effective coping mechanisms early in their diagnosis [54,55].

Another study found that patients with relapsing-remitting (RR) MS courses and higher depression scores adopted avoiding strategies more frequently [23]. Depression, stress frequency, trait anxiety, and mental health QoL were influenced by both adaptive and maladaptive coping styles. Particularly, the severity of stressful events was mainly predicted by maladaptive coping styles. Depression and mental health QoL had strong ties to how coping strategies were employed. Specifically, emotional preoccupation and venting were linked to worse psychosocial outcomes, while positive reinterpretation and growth were associated with more favorable results [43].

**Social and family support**

Individuals with adequate social support and functional family relationships showed improved perceptions of QoL and its various aspects. Patients who received social support demonstrated higher levels of active coping, planning, and both emotional and instrumental support use, all of which correlated with improved QoL. Specifically, those supported mainly by family and friends exhibited better social relationships scores and more effective employment of coping strategies [45]. Unexpectedly, longer disease duration is associated with reduced family support, regardless of the level of disability. Families might feel overwhelmed by the persistent and unchanging nature of MS, leading them to distance themselves as a coping mechanism [56]. While actual support from family might not decrease, patients could perceive it as less due to increasing impairments. Additionally, higher levels of education are linked to reduced family support, which could be due to more extensive educational experiences leading to weaker family connections and a broader social network [57].

Marital and sexual relationships are protective factors against physiological problems [58]. Women who use coping strategies for their illness experience lower stress levels, better relationship-building success, and higher satisfaction and mental health [59]. These benefits extend to all aspects of personal and social life, including successful sexual relationships, sexual health, and satisfaction. Strengthening problem-focused coping strategies indirectly enhances sexual satisfaction [41,43]. Other studies on Iranian women with MS have highlighted the influence of demographic factors, such as education and employment status, and the supportive role of husbands in enhancing sexual function [60,61] with positive effects on QoL [62].

Patients who have closer relationships with family and friends, as well as more satisfying sentimental and sexual lives, tend to score higher in problem-focused coping strategies, adopting a more active approach to dealing with stressors [38].

**Stage and severity of disease**

Coping styles do not depend on the type of MS, and the severity of the disease has little influence on the choice of the coping styles [36]. However, coping strategies vary depending on the stage of the illness. According to the literature [63], numerous studies [32] have found that problem-solving but also emotional release and avoidance are adaptive coping strategies during the initial years following an MS diagnosis. Early emotional responses typically include anger, anxiety, and depression [64,65]. The findings of the study of Calandri [52] indicated that expressing emotions and discussing feelings with others are effective strategies for combating depression in newly diagnosed patients. Additionally, the role of avoidance as an adaptive strategy is consistent with studies on patients with prolonged disease duration [15,66], and it is recognized as beneficial even in the early stages of the illness. In the initial years post-diagnosis, as patients adjust to their new reality, avoidance may help them manage the uncertainty of their future and cope with symptoms that are difficult to change [67].

Several studies have linked the severity of physical disability with the use of specific coping strategies. Patients with a current moderate to severe physical disability (EDSS > 2) were found to have utilized more emotional discharge strategies at the time of diagnosis compared to those with a lower current physical disability [39]. Further, the physical component of QoL was more influenced by disease severity than by coping strategies [40].

Interestingly [48] it was shown that high approach coping and low avoidant coping were associated with higher positive affect and well-being (PAWB), but only when symptoms were low. The beneficial effects of positive coping strategies diminished as pain and fatigue increased. These findings align with the Dynamic Model of Affect (DMA), suggesting that under low-stress conditions (e.g., days with less severe pain/fatigue), negative and positive affect are relatively independent, indicating high affective complexity [68].

**Sociodemographic variables and personality traits**

Although there is no consensus in the literature, demographical variables significantly affect coping strategies. Particularly, younger age correlates with maladaptive coping strategies [41]. This is in line with Holland’s [69] findings, which noted that younger individuals are more likely to adopt maladaptive coping strategies, such as humor, focusing on emotions, and substance use. In contrast, other researchers found no correlation between age and coping strategies [52].

Female gender is positively associated with using religion as an emotion-focused coping strategy in MS [44]. This is consistent with findings from Holland et al. [69] and Zengin et al. [45], who noted that women use religion more frequently than men. Older individuals tend to use religion and self-blame more, while those with higher education levels are more inclined towards planning and seeking emotional support. Higher education may enable MS patients to select more effective and adaptive strategies and better utilize social support.

Rommer et al. [70] reported that female MS patients often search for information and exchange experiences. Patients with higher levels of education were more inclined to use problem-focused coping strategies for managing or resolving stressors by seeking information, support, guidance, and engaging in alternative activities to foster new satisfaction sources [38]. These findings align with those reported by Goretti and colleagues [23].

Personality traits also affect the capacity to deal with stressful situations. Although the duration of the disease does not seem to impact personality traits [50], it has been shown that patients with relapsing remitting multiple sclerosis (RRMS) who exhibit higher levels of neuroticism and passive coping strategies tend to have reduced QoL. Conversely, those with RRMS who display greater levels of extroversion, openness, and agreeableness experience enhanced QoL and improved physical and mental health [50]. Recent research in Austria and Italy also indicates a correlation between neuroticism and lower QoL among MS patients [71,72].

**Positive or negative coping strategies and quality of life**

Nada [51] reported that MS patients exhibited fewer problem-focused coping strategies and less positive reinterpretation compared to controls. This aligns with previous studies [73,74] indicating that MS patients often show poorer adjustment across various dimensions [75]. Patients with lower psychological well-being and QoL scores, particularly related to coping and perceptions of rejection, scored higher in emotion-focused strategies [38]. Wilski et al. [37] supported the notion that emotion-focused coping is maladaptive and negatively associated with HRQoL [63,76]. Despite some researchers suggesting potential benefits of emotional coping [77,78], the study by Goretti [23] did not find evidence supporting these positive effects. Another critical finding is that avoidance coping is generally considered maladaptive, negatively impacting HRQoL in MS patients. However, some studies suggest a protective aspect of avoidance strategies, especially when patients have little control over their situation. Wilski et al. [37] found that increased use of avoidance strategies could be beneficial, particularly in the physical dimension of HRQoL, by not confronting physical difficulties and avoiding unmodifiable symptoms. Dennison et al. [66] highlighted that an avoidant and resistant approach to illness might help adjust in certain areas.

Additionally, it was shown for the first time that a strong sense of coherence is crucial for patients’ adjustment, mediating the beneficial effects of emotional and avoidant coping strategies. One possible explanation for the positive correlation between emotional release and a sense of coherence is that expressing emotions helps patients better understand their experiences and manage their feelings [52]. Patients who employed positive coping strategies, such as planful problem-solving, positive reappraisal, and seeking social support, demonstrated significantly higher QoL scores and experienced greater levels of social support [47]. The longitudinal study conducted by McCabe et al. [32] revealed the detrimental impact of wishful thinking and the beneficial impact of maintaining a positive focus on QoL in MS patients. This research underscores the broad influence of coping strategies across diverse QoL domains, not only in managing illness-related issues but also in dealing with other life aspects.

### 4.1. Study Strengths and Limitations

The reviewed studies have several limitations. Many studies employ a cross-sectional design, which is not suitable for detecting potential relationships between coping strategies and QoL. The absence of control groups or the presence of selection bias further undermines the validity of the findings. Self-selection of participants, a common research bias, is also noted [52]. Moreover, one study reports non-random participant selection and a heterogeneous sample [44]. There is also considerable heterogeneity in the measurement instruments used to assess coping strategies and QoL, as well as variability in patient populations [38].

The strength of this review is that it provides a clear overview of the coping strategies used by MS patients at various stages of the disease, taking into account numerous factors such as demographics, emotional variables, personality traits, and family support. This work can therefore serve as a guide for therapists to structure specific and personalized training to promote effective coping strategies, resulting in positive effects on QoL.

### 4.2. Future Directions

Future studies should further investigate cognitive performance evaluation, as it could significantly impact coping strategies. Various tests, like the Mini-Mental State Examination (MMSE) and the Brief Repeatable Battery of Neuropsychological Tests (BRB-N), have been used in different studies to assess this construct. Despite cognitive dysfunction being present in about 50% of MS patients [79] and its strong association with deficits in everyday functioning [80,81], few studies [49,51,54] have explored the relationship between cognitive dysfunction and coping. Cognitive dysfunction in MS can be seen as a stressor, as previously suggested [81]. Since coping involves cognitive processes, cognitive dysfunction may directly affect an individual’s ability to implement effective cognitive and behavioral coping strategies.

Given the significant impact of coping strategies on the QoL for individuals with MS, it is essential to implement targeted rehabilitation strategies. Additionally, it is also important to include specific tests that assess the relationship between QoL and coping strategies [82]. Psychoeducation for family members can be crucial, helping them understand how to provide better support to MS patients [83]. Educating families can alleviate the burden on patients and promote more effective coping mechanisms [84]. Additionally, therapists should focus on the emotional aspects of MS. Addressing emotional health through targeted therapy can improve psychological well-being, reduce depression and anxiety, and enhance overall QoL [85]. One specific approach that has shown promise is mindfulness, which can be an effective coping strategy for MS patients [86]. This holistic approach, involving both patients and their support networks, can lead to more resilient and adaptive coping strategies, ultimately improving the long-term outcomes for those living with MS.

## 5. Conclusions

In conclusion, this review highlights the critical role of coping strategies in managing MS and their impact on patients’ QoL. The studies examined show that adaptive coping mechanisms, demographic factors, and personality traits significantly influence how individuals with MS cope with their condition. Task-oriented and problem-focused coping strategies are prevalent and associated with better QoL outcomes, while emotion-focused and avoidance strategies are generally linked to poorer QoL, except in specific contexts where avoidance provides temporary relief. This work underscores the importance of incorporating psychoeducational and therapeutic interventions that focus on emotional health, social support, and tailored coping strategies to improve the long-term outcomes for individuals with MS. Further research is needed to explore the dynamic interactions between coping strategies and various QoL domains over time, providing a more comprehensive understanding of how to best support MS patients in managing their disease.

## Figures and Tables

**Table 1:** Main characteristics of the included studies.

Study	Study Design	Population	Disease Severity	EDSS	Education	Type of MS	Coping Test	QoL Test	Emotion Test	Cognitive Test	State	Results
Brands [36]	Cross-sectional study	310 (228 females) Mean age: 49 (SD 10.3)	Illness duration 9.5	Mean 3.7	Low: 77, medium: 109 High:123	RRMS 209; PPMS 31; SPMS 68	CIIST-T; CCS-EC	LiSat-9	HADS	NA	South of Netherlands	CISS-A was positively associated with QoL
Calandri [52]	Cross-sectional study	102 (63 females) M:35.8; SD: 11.9	Mean disease duration 1.6 (SD: 0.8)	Score between 1 and 4	Middle school diploma: 20; High school diploma: 59; Degree: 23	RRMS 97; PPMS 1; SPMS 4	CMSS	SF-12 Health Survey	SOC; PANAS	NA	Italy	Problem-solving emotional release and avoidance can be adaptive coping strategies for recently diagnosed MS patients
Cerea [39]	Cross-sectional study	108 (84 female) mean age 38.09 (SD = 3.24)	Mean disease duration 6.35	Median score 1.5	14.31 years	99/108 RRMS; 9/108 SPMS	CRI-Adult	MSQoL-29	DASS-21	NA	Italy	Problem solving strategies positively impacted mental HRQoL
Carnero-Contentti [41]	Cross-sectional study	249 (186 female) 38.6 (±10.7)	MS duration: year 7.3 ± 6.5	Mean 1.98 ± 1.8	No education: 32; Primary school: 61; High school: 64; Tertiary education University: 92	RRMS	COPE-28	MSIS-29	FSS	NA	Argentina	Maladaptive coping strategies are associated with worst QoL
Corallo [49]	Observational study	88 divided in two groups (injecting group 44 = age 48.30 ± 13.14; oral group: 44 = age 48.45 ± 12.68 years)	Injection group: 15.21 ± 8.3 Oral group: 12.83 ± 8.20	NA	Injecting group: 11.33 ± 3.77 Oral group: 11.55 ± 3.12	NA	COPE	MSQL-54	Morisky Medication Adherence Scale	BRB-N	Italy	A correlation between therapeutic adherence, adaptive coping strategies, and mental health in the injective MS group
Farran [47]	Pilot Study	34 (56% female) 36± 11	Disease duration 9 ± 8 years	NA	NA	RRMS: 64.71; PPMS: 5.88; SPMS: 11.76; PRMS: 2.94; Patient did not know: 14.71	WOCQ	MusiQoL	BDI-II; BAI; FSS; SPS.	NA	Lebanon	Positive coping strategies are associated with better QoL and lower psychological distress, while negative strategies, particularly escape avoidance, are linked to poorer outcomes.
Gil-González [44]	Longitudinal study	314 (213 female) mean age 45.31 years (±10.77),	Months since diagnosis 145.68	3.17 ±1.92	Primary education: 44 Secondary education: 102 University of higher: 168	Remittent 272 (86.6) Progressive: 42 (13.4)	COPE-28	SF-12	MPSS	NA	Spain	Reducing dysfunctional coping strategies and promoting cognitive reframing may improve HRQOL in individuals with MS.
Goretti [23]	Observational study	104 (72 female) 45.3 ± 10.9 years	Mean disease duration 17.9 ± 13.2,	mean EDSS 2.8 ± 2.0	mean education 12.1 ± 3.1,	73 patients RRMS, 26 SPMS, 5 PPMS	COPE-NVI	MSQOL-54	BDI, STAI-Y, EPQ, FSS	NA	Italy	MS people use more frequently avoiding strategies. Depression and anxiety impact negatively QoL
Grech [43]	Cross-sectional study	107 (83 female) 48.80 ± 11.10	Time since diagnosis 9.82 ± 7.46	2.90 ± 2.31	Secondary 30 (28.04); College 21 (19.63); Undergraduate 34 (31.77); Postgraduate 22 (20.56)	RRMS: 83; SPMS 24	60-item COPE inventory	MSQOL-54	BDI, STAY, Daily Hassles Scale,	NA	Australia	Depression, stress frequency, trait anxiety, and mental health QOL were influenced by both adaptive and maladaptive coping style
Hernandez-Ledersma [46]	Cross-sectional study	26 mean age 39.2 ± 10.6 years	Mean age at diagnosis was 32.7 ± 9.7 years	NA	Completed Middle school: 11.5%; High school diploma: 19.2%; Technical degree: 7.7%; College degree: 57.7%; Postgraduate degree: 3.8%	RRMS: 50%; PPMS: 11.5%; SPMS: 7.7%; Unclassified MS: 30.8%	Spanish version of CSI	WHOQOL-BREF	BDI, BAI, FSS, FF-SIL, Duke-UNC-11	NA	Mexico	Positive coping strategies, along with a supportive psycho-social environment and good physical health, improve QoL perception.
Krstić [50]	Cross-sectional study	66 (34 female), age 41.6 ± 7.1	Duration of illness 8.1 ± 5.1	2.4 ± 1.1	8–12 years: 4 patients (6%); 12–16 years: 46 patients (70.1%); More than 16 years: 16 patients (23.9%)	RRMS	CSI	MSQOL-54	NEO-PI-R	NA	Serbia	Higher neuroticism and passive coping strategies reduce the QoL
McCabe [32]	Longitudinal study	Time 1: 381 MS people (237 female, mean age = 45.18 years). 291 HP; Time 2: 283 (186 female) and 239 HP	NA	NA	NA	NA	WOCQ	WHOQOL	NA	NA	Australia	Social support, focusing on the positive, and wishful thinking predict QOL
Mikula [40]	Cross sectional study	113 (87 female) 40.82 ± 9.22	Disease duration 8.40	3.31 ± 1.37	NA	RRMS 96; SPMS 17.	CSE	SF-36	NA	NA	Slovakia	Managing unpleasant emotions and thoughts, play a crucial role in improving the mental well-being of MS patients.
Montel [42]	Cross-sectional study	135 (66 female) 44.3 (11.8)	Disease duration 8.7 (6.8)	Mean score 3.8. RRMS: 1.8; SPMS: 5.4; PPMS: 4.6.	NA	53 RRMS; 53 SPMS; 29 PPMS	WCC; CHIP;	SEP 59	MADRS; EHD; HAMA;	MINI; FAB	France	SPMS patients tend to rely heavily on emotional coping strategies, whereas PPMS patients utilize more instrumental strategies.
Nada [51]	Prospective case-control study	40 (24 female) 33.8 ± 8.91; 20 HC (12 female)	6.67 ± 4.03	PPMS: Mean EDSS = 5.9 ± 1.2; RRM: Mean EDSS = 4.3 ± 0.8; Total MS group: Mean EDSS = 4.96 ± 1.12	NA	22 RRMS; 18 PPMS	Coping processes Scale, EPQ	QoL	HADS	MMSE	Egypt	Exercising restraint and positive reinterpretation have proven to be more effective in enhancing patients’ QoL
Vanotti [38]	Cross-sectional study	90 (59 female) mean age 40.97 ± 12.85	Disease evolution 10.76 ± 9.72 years	2.48 ± 1.79	13.46 ± 3.93	RRMS: 95.56%; PPMS: 2.22%; SPMS: 2.22%.	CRI-A	MusiQol	BDI, Fatigue severity scale	NA	Argentine	Emotion-focused coping strategies were negatively correlated with QoL
Wilski [37]	Cross-sectional study	382 (256 female) 46.4 ± 11.9 (18–82)	NA	Mean EDSS Score: 4.4 ± 1.7 (range: 1–8.5)	Primary/Vocational: 25.1%; Secondary: 43.2%; Higher: 31.7%	RRMS: 158; PPMS: 88; SPMS:70; PRMS: 33; Unknown type: 33.	CISS	MSIS-29	NA	NA	Poland	Younger MS patients with higher acceptance, using problem-solving and avoidance coping strategies, had longer disease duration and better HRQoL.
Zengin [45]	Cross-sectional study	214 (126 female)	NA	NA	Primary school and less: 31; Secondary school: 26; High school: 64; Bachelor’s degree: 67; Postgraduate: 20	NA	COPE	WHOWOL-BREF	NA	NA	Turkey	Problem-focused coping strategies were positively correlated with QoL
Valentine [48]	Cross-sectional study	102 average age 44.68 years.	Average MS disease duration was 9.26 years (SD = 8.25 years).	NA	Primary school and less: 16.8%; Secondary school: 12.1%; High school: 29.9%; Bachelor’s degree: 31.3%; Postgraduate: 9.3%	NA	COPE	PAWB	NA	NA	Turkey	Problem-focused coping strategies were positively correlated with QoL

Legend: RRMS = relapsing remitting multiple sclerosis; PPMS = primary progressive multiple sclerosis; SPMS = secondary progressive multiple sclerosis; LiSat-9 = Life Satisfaction Questionnaire; CISS-T = Coping Inventory for Stressful Situations (CISS-T, task-oriented; CISS-E, emotion-oriented; CISS-A, avoidance); HADS = Hospital Anxiety and Depression Scale; CMSS: Coping with Multiple Sclerosis Scale; SOC: Sense of Coherence; PANAS: Positive Affect Negative Affect Schedule; MSQoL-29 = Multiple Sclerosis Quality of Life-29; CRI-Adult = Coping Responses Inventory-Adult form CRI-Adult. DASS-21 = Depression Anxiety Stress Scale-21; HRQoL = Health related quality of life; FSS = Fatigue Severity Score; COPE = Brief coping orientation to problems experienced; MSQL-54 = Quality of Life-54; BRB-N = Brief Repeatable Battery of Neuropsychological Tests; SF-12: 12-Item Short Form Health Survey. MPSS = Multidimensional Scale of Perceived Social Support; BDI = Beck Depression Inventory; STAI-Y = State-Trait Anxiety Inventory; FSS = Fatigue Severity Scale; WHOQOL-BREF = the World Health Organization Quality of Life Questionnaire; FSS = Krupp’s fatigue severity scale; FF-SIL = Family functionality; Duke-UNC-11 = Social Support; NEO-PI-R = Revised NEO Personality Inventory; HP = Healthy population; MINI = Mini International Neuropsychiatric Interview; MADRS = Montgomery and Asberg Depression Rating Scale; EHD = Depressive Mood Scale; HAMA = Hamilton Anxiety; FAB = Frontal Assessment Battery; WCC = Ways of Coping Checklist; CHIP = Coping with Health, Injuries, and Problems scale; MMSE = Mini-Mental State Examination; MusiQol = Multiple Sclerosis International Quality of Life questionnaire; CRI-A = Coping response inventory; MSIS-29 = Multiple Sclerosis Impact Scale-29; PAWB = Positive Affect and Well Being Short Form; CSI = Coping Strategies Inventory; SF-36 = 36 items Short-Form; BAI = Beck Anxiety Inventory; SPS = Social Provisions Scale.

## Data Availability

Not applicable.
